# Targeting pancreatitis blocks tumor-initiating stem cells and pancreatic cancer progression

**DOI:** 10.18632/oncotarget.3499

**Published:** 2015-03-26

**Authors:** Altaf Mohammed, Naveena B. Janakiram, Venkateshwar Madka, Misty Brewer, Rebekah L. Ritchie, Stan Lightfoot, Gaurav Kumar, Michael Sadeghi, Jagan Mohan R. Patlolla, Hiroshi Y. Yamada, Zobeida Cruz-Monserrate, Randal May, Courtney W. Houchen, Vernon E. Steele, Chinthalapally V. Rao

**Affiliations:** ^1^ Center for Cancer Prevention and Drug Development, Department of Medicine, Hem-Onc Section, PC Stephenson Cancer Center, University of Oklahoma Health Sciences Center, Oklahoma City, OK, USA; ^2^ Department of Cancer Biology, University of Texas, MD Anderson Cancer Center, Houston, TX, USA; ^3^ Digestive Diseases Section, Department of Medicine, University of Oklahoma Health Sciences Center, Oklahoma City, OK, USA; ^4^ Division of Cancer Prevention, Chemoprevention Agent Development Research Group, National Cancer Institute, Bethesda, MD, USA

**Keywords:** pancreatic cancer, inflammation, dual COX-5-LOX inhibition, cancer stem cells, p48^Cre/+^-LSL-Kras^G12D/+^ mice

## Abstract

Recent development of genetically engineered mouse models (GEMs) for pancreatic cancer (PC) that recapitulates human disease progression has helped to identify new strategies to delay/inhibit PC development. We first found that expression of the pancreatic tumor-initiating/cancer stem cells (CSC) marker DclK1 occurs in early stage PC and in both early and late pancreatic intraepithelial neoplasia (PanIN) and that it increases as disease progresses in GEM and also in human PC. Genome-wide next generation sequencing of pancreatic ductal adenocarcinoma (PDAC) from GEM mice revealed significantly increased DclK1 along with inflammatory genes. Genetic ablation of cyclo-oxygenase-2 (COX-2) decreased DclK1 in GEM. Induction of inflammation/pancreatitis with cerulein in GEM mice increased DclK1, and the novel dual COX/5-lipoxygenase (5-LOX) inhibitor licofelone reduced it. Dietary licofelone significantly inhibited the incidence of PDAC and carcinoma *in situ* with significant inhibition of pancreatic CSCs. Licofelone suppressed pancreatic tumor COX-2 and 5-LOX activities and modulated miRNAs characteristic of CSC and inflammation in correlation with PDAC inhibition. These results offer a preclinical proof of concept to target the inflammation initiation to inhibit cancer stem cells early for improving the treatment of pancreatic cancers, with immediate clinical implications for repositioning dual COX/5-LOX inhibitors in human trials for high risk patients.

## INTRODUCTION

Despite tremendous scientific effort for over three decades, pancreatic cancer (PC) remains a devastating, almost uniformly lethal disease with < 5% five-year survival. Current strategies for management of PC patients lack appreciable benefit. Gemcitabine, the current drug choice for pancreatic ductal adenocarcinoma (PDAC) treatment marginally increases the survival by about few weeks. Recent studies demonstrate that pre-invasive precursors progress slowly over many years to decades to development of invasive PCs [1–3]. Thus, there is a time frame of several years for effective intervention strategies. Interventions that can delay or inhibit the progression of precursor lesions to PC should drastically improve the overall survival rate. Premalignant pancreatic intra-epithelial neoplasia (PanIN) lesions are the most common precursors to invasive PDAC, rendering them promising targets for intervention, especially in the high-risk population [4]. Human PDAC (> 90%) often is characterized by activating mutations in KRAS oncogenes [5, 6]. Recently, several genetically engineered mouse models (GEMs) of PC have been developed that recapitulate human disease progression. Mice harboring a conditional K-ras mutant allele (LSL-Kras-^G12D/+^) in combination with a pancreas-specific Cre recombinase transgene (Pdx^Cre/+^ or p48^Cre/+^) develop a full range of PanIN lesions in the pancreas before succumbing to invasive PDAC and other tumors at late ages [3, 7]. It also was demonstrated recently that the KrasG12D-dependent mouse model of PDAC that accurately mimics the therapeutic response of human PDAC offers the opportunity to develop novel treatments [3, 9–11]. We have shown that these GEM are excellent models for understanding PanIN progression and are useful for drug and other intervention studies [3, 12–18].

Inflammation is evident at the earliest stages of cancer progression and is capable of fostering growth and progression of early lesions to PDAC and into metastatic cancers. Chronic pancreatitis is considered to be a risk factor for pancreatic tumor growth [19, 20]. Initiation of pancreatic lesions and their progression to PDAC and further metastatic invasion also are associated with inflammation. Among various inflammatory modulators, eicosanoid-derived molecules such as prostaglandin (PG) E_2_ (PGE_2_) and leukotrienes (LTs) have been shown to modulate tumor progression; and use of non-steroidal anti-inflammatory agents that block PGE_2_ has been shown to inhibit tumor growth [15, 21–23]. Thus, preclinical and clinical data underscore the importance of arachidonic acid metabolism-related inflammatory responses in pancreatic tumorigenesis.

Several lines of evidence show that cyclooxygenase-2 (COX-2) and 5-lipoxygenase (5-LOX) are over-expressed significantly in cancers. Preclinical studies show that COX-2 inhibitors may inhibit pancreatic cancer both *in vitro* and *in vivo*; however, those studies have not been translated for clinical usage [15, 21, 24]. Morevover, use of celecoxib, a COX-2 inhibitor, in the prevention of adenomatous polyps was associated with a significant increase in risk of cardiovascular (CV) events [25]. In another study, the relative risk of CV events with the use of celecoxib as compared with placebo was 1.30 [26]. It is widely accepted that selectively blocking COX-2 will shift arachidonic acid metabolism towards the 5-LOX pathway, overproducing leukotrienes and leading to increased prothrombotic effects [25, 27–30]. Cancer Stem cell (CSC) populations have been identified in a variety of human cancer types and recent evidence suggests that the COX-2 metabolite PGE_2_ enhances stemness and proliferation of CSCs [31, 32]. In pancreatic carcinogenesis, increased inflammation and tumor cell stemness not only contribute to progression of tumor growth and invasion, but also to resistance to chemotherapy. Thus, in this study we evaluated the potential role of inflammation on CSC markers during PC progression and the use of the novel anti-inflammatory dual COX/5-LOX inhibitor licofelone in PC arising de novo in GEMs.

## RESULTS

### Activation of inflammation and CSCs during progression of pancreatic cancer

**Figure 1:**
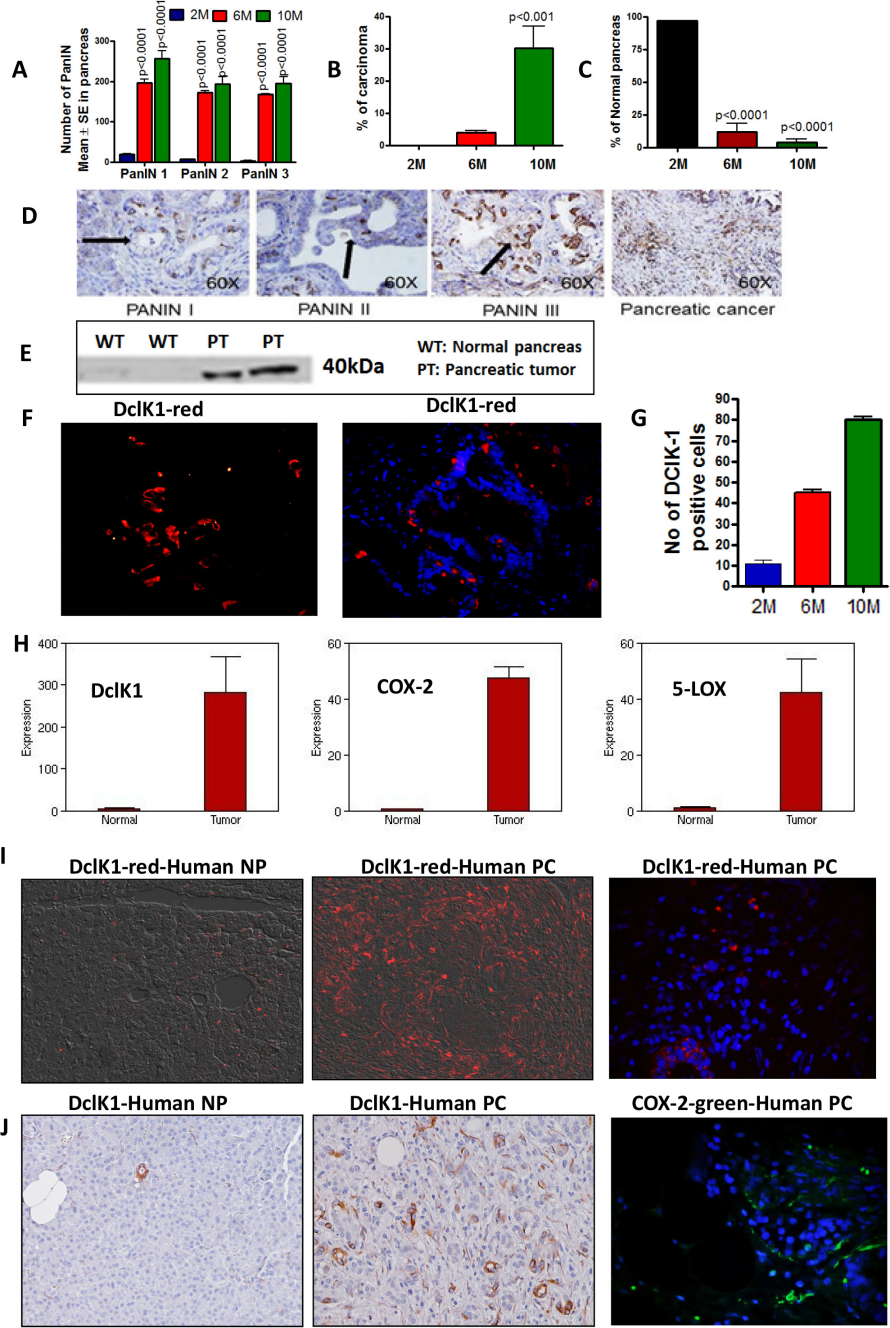
Activation of inflammation and CSCs during progression of pancreatic cancer **A–C.** Histopathological analysis of pancreas from 2-, 6- and 10-month-old GEM mice using H&E staining. Pancreas from animals showing PanIN lesions **A.** carcinoma **B.** and normal pancreas **C. D–G.** Expression of DclK1 in PanINs and PDAC. **D.** IHC for DclK1 in PanINs and PDAC, **E.** Western blotting of normal pancreas vs pancreatic tumor for DclK1 expression, **F.** IHF showing DclK1 (red) (left panel) and IHF showing DclK1 (red) merged with DAPI (blue) (right panel). **G.** Number of DclK1 positive cells in 2-, 6- and 10-month-old GEM. **H.** Whole genome transcriptome analysis by Solid sequencing showing increased mRNA expression of DclK1, COX-2 and 5-LOX in pancreas from GEM mice compared with wild type mice. **I.** IHF showing DclK1 (red) and COX-2 (green) expression in human normal pancreas (NP) and pancreatic ductal adenocarcinoma (PC). **J.** IHC showing DclK1 (brown) and IHF showing COX-2 (green) expression in human pancreatic ductal adenocarcinoma.

p48^Cre/+^-LSL-Kras^G12D/+^ mice were analyzed for the expression of COX-2, 5-LOX and DclK1 (Fig. 1, Supplementary Fig. 1). Inflammation is the first step during the initiation and progression of PC. As the mice aged from 2 to 6 to 10 months, along with progression of PanIN lesions and carcinoma, we observed a significant increase in the inflammatory COX-2 along with proliferating cells (Fig. 1A–1C, Supplementary Fig. 1). Similarly, DclK1 was expressed at the early stage, i.e., in 2 month-old GEM, and was seen in all of the pancreatic precursor lesions (PanIN1, PanIN2 and PanIN3) (Fig. 1D). DclK1 expression increased linearly as the disease progressed to PDAC (Fig. 1E–1G). Genome-wide transcriptome analysis of normal pancreas and ductal adenocarcinoma revealed that the mRNAs of COX-2, 5-LOX and DclK1 were increased significantly (up to 53-fold; *p* < 0.01–0.001) in the PDAC (Fig. 1H). Also, we found high expression of DclK1 and COX-2 in human PDAC (Fig. 1I & 1J). These results strongly indicate that inflammation and stem cell regulation occur at the initial stages of PC and progress simultaneously as the diseases lead to the PDAC stage.

### Genetic ablation of COX-2 inhibits formation of DclK1 cells early during tumorigenesis in GEM

**Figure 2:**
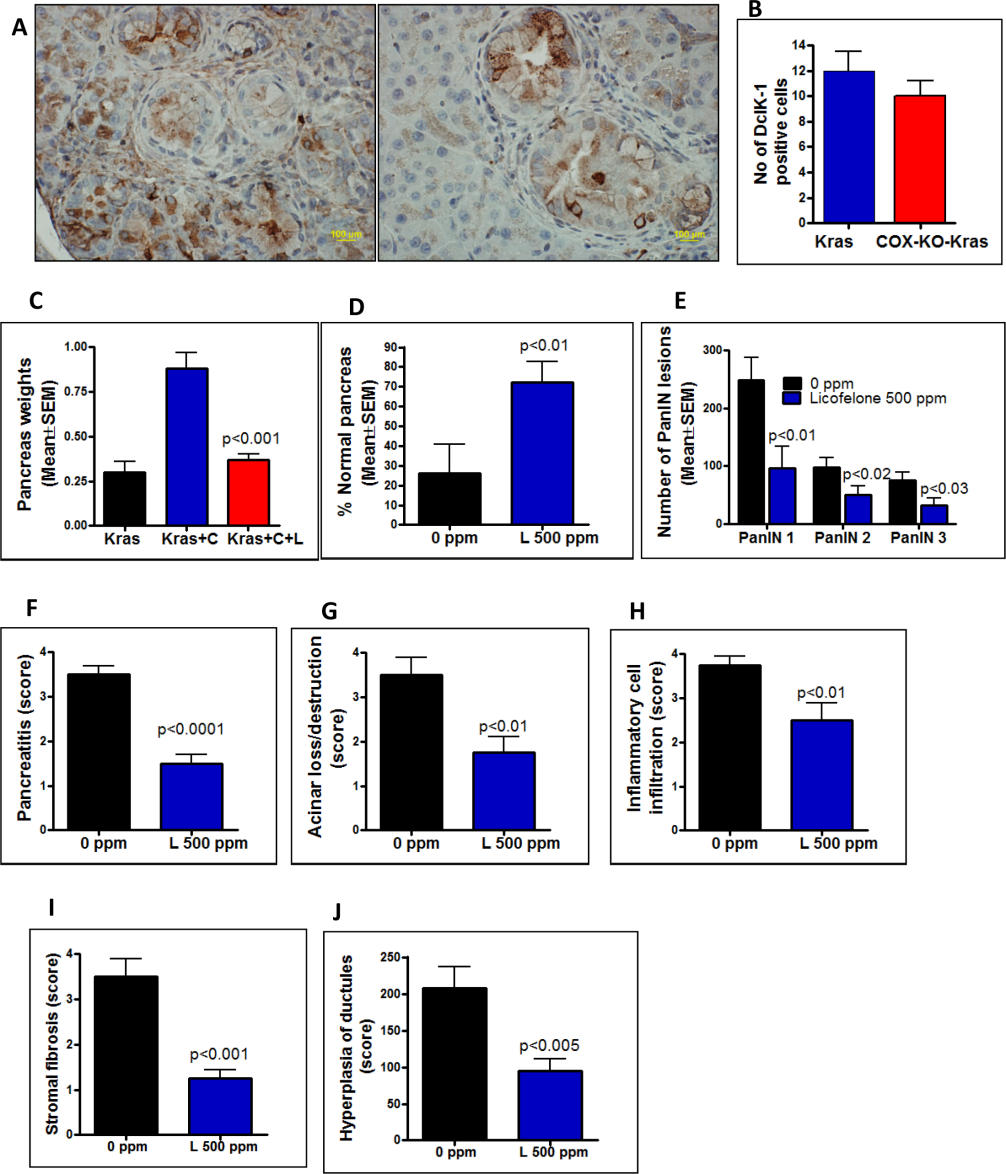
A–B. Effect of genetic ablation of COX-2 on DclK1 expression Decreased expression of Dclk1 was observed in the COX-2 knock-out GEM mice (A, right panel) compared with GEM mice alone (A, left panel) as was a decreased number of DclK1-positive cells **B. C–J.** Histopathological analysis using H&E staining of pancreas from cerulean (C)-treated GEM mice with and without licofelone (L) in the diet. **C.** Effect of licofelone on pancreas weight at the termination of the experiment. Licofelone significantly reduced the pancreatic tumor weights. **D–E.** Effect of licofelone on the percentage of normal-appearing pancreas and on PanIN multiplicity. **F–J.** Compared with untreated mice, the licofelone-treated GEM mice showed decreased: **F.** pancreatitis, **G.** acinar destruction, **H.** inflammatory cell infiltration, **I.** stromal fibrosis, **J.** hyperplasia of ductules.

To determine whether inflammation is a key factor driving tumorigenesis through CSCs, we used the KrasG12D GEM (LSLKras/Ela-CreERT mice) alone or crossed with COX2 conditional knockout mice (COXKO/LSL-Kras/Ela-CreERT) to study the effect of COX-2 ablation on DclK1. We observed a moderate inhibition of DclK1 upon deletion of COX-2 in four week-old GEM mice (Fig. 2A–2B). It is well known that when COX-2 is inhibited, a shift in arachidonic acid metabolism occurs, leading to 5-LOX proinflammatory activities. Hence further studies using a dual COX-5-LOX model is warranted to evaluate the role of this shift in inflammatory mediators on DclK1 cells.

### Licofelone inhibits inflammation induced DclK1 by pancreatitis in GEM

We investigated whether CSC DclK1 is regulated directly upon induction of inflammation with cerulein and whether treatment with the anti-inflammatory dual COX-LOX inhibitor licofelone effectively blocks the DclK1 increase in p48^Cre/+^-LSL-Kras^G12D/+^ GEM (Supplementary Fig. 2A–2C). Pancreas weights in the p48^Cre/+^-LSL-Kras^G12D/+^ GEM were increased with the inflammatory conditions and significantly reduced upon licofelone treatment (Fig. 2C–2D). Histological analysis showed 100% penetrance of pancreatic precursor PanIN lesions in the GEM (Fig. 2E). The numbers of PanIN 1, PanIN 2, and PanIN 3 lesions in the GEM were (means ± SE): 248 ± 39, 98 ± 16, and 75 ± 14, respectively; in the licofelone treated GEM, PanIN 1, PanIN 2, and PanIN 3 numbers were 96 ± 38, 50 ± 15 and 32 ± 12, respectively (Fig. 2E). The number of PanIN 3 lesions or carcinoma *in situ* was decreased by ~3-fold in the licofelone-treated mice (Fig. 2E). A significant decrease in the number of PanIN 1 and PanIN 2 lesions also was observed in pancreas of licofelone treated GEM.

We observed mild pancreatitis in the licofelone-treated mice via histopathology whereas in the untreated GEM, pancreatitis was moderate to severe (Fig. 2F). About 10–30% acinar destruction was found in the treatment group whereas up to 50% was found in the untreated mice (*p* < 0.01, Fig. 2G). Significantly decreased inflammatory cell infiltration and stromal fibrosis were observed in the licofelone treated mice (Fig. 2H, 2I). More than a two-fold increase in hyperplasia of ductules was noticed in the pancreata of untreated mice compared with those of licofelone treated mice (Fig. 2J). Supplementary Table 1. shows the scoring patterns for cerulein treated mice. However, no pancreatitis was seen in the pancreata of either untreated or licofelone-treated mice not treated with cerulein.

**Figure 3:**
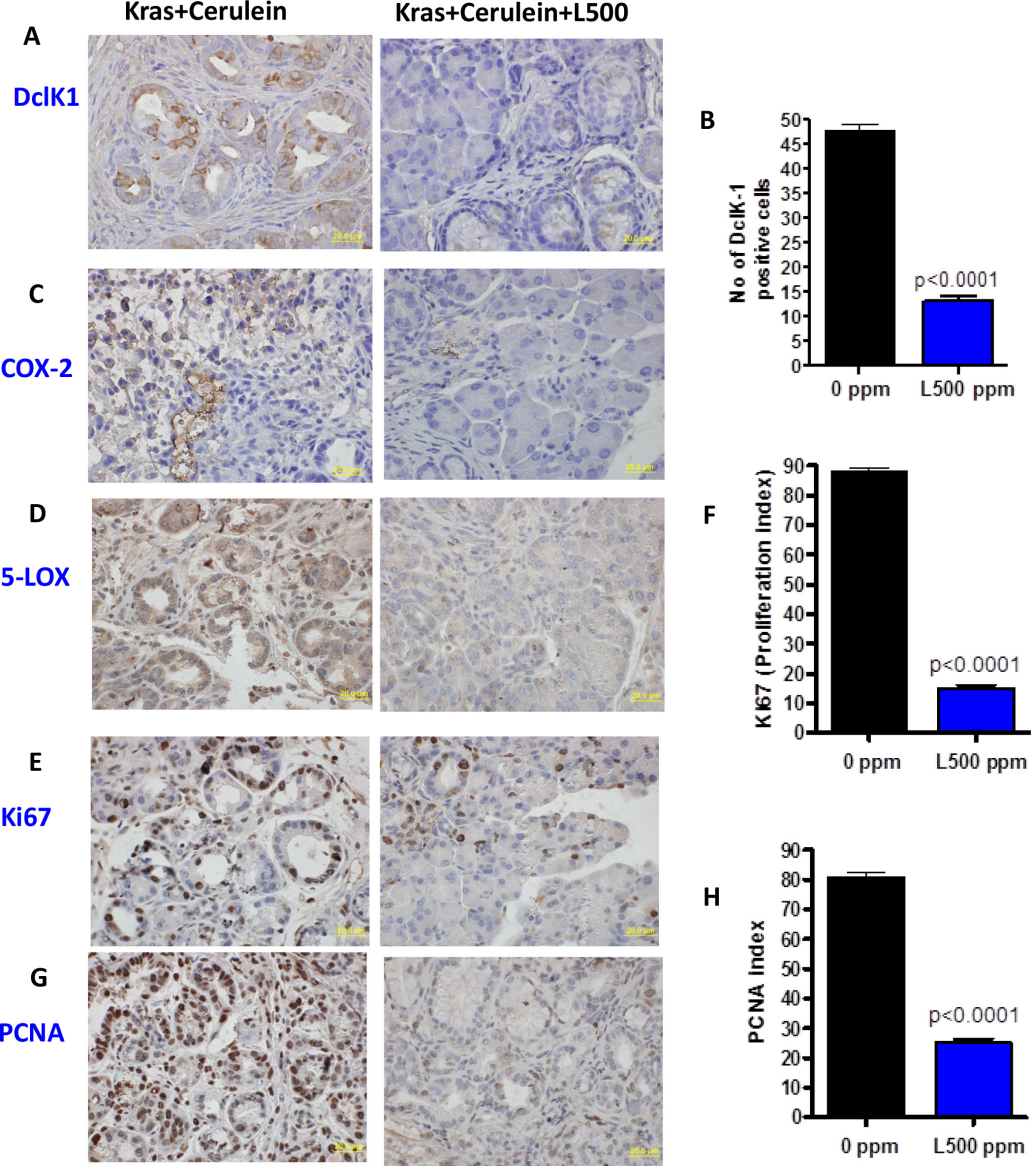
A–H. Effect of licofelone on cerulean-induced pancreatitis on inflammation, proliferation and DclK1 expression Twelve week old p48^Cre/+^-LSL-Kras^G12D/+^ mice (*N* = 6/group) were injected with 5 μg/mice of cerulein every day for six consecutive days. Licofelone (500 ppm) was fed to a group of mice starting one day before cerulein injection to one day after the last cerulein injection. One day after cerulein injection, all mice were euthanized by CO_2_ asphyxiation and necropsied. Pancreata were collected from all groups, weighed and snap frozen in liquid nitrogen for further analysis. Immunohistochemical analyses were performed with paraffin-embedded and micro-sectioned pancreatic tissues as described in the Methods section. A significantly decreased expression of DclK1, COX-2, 5-LOX, PCNA and Ki67 was seen in licofelone-treated GEM. A significantly reduced number of DclK1 positive cells and proliferation index was seen in the licofelone-treated GEM compared with untreated mice.

A marked increase in number of DclK1 cells was observed in the cerulean-induced inflammation GEM mice (~3 months old) (means ± SE; 48 ± 13), comparable to the number of DclK1 cells in non-cerulein treated mice at 6 months of age. Licofelone treatment inhibited DclK1 cells, inflammation and proliferation significantly (Fig. 3).

### Pharmacological inhibition of inflammation by licofelone inhibits tumor development in GEM

**Figure 4:**
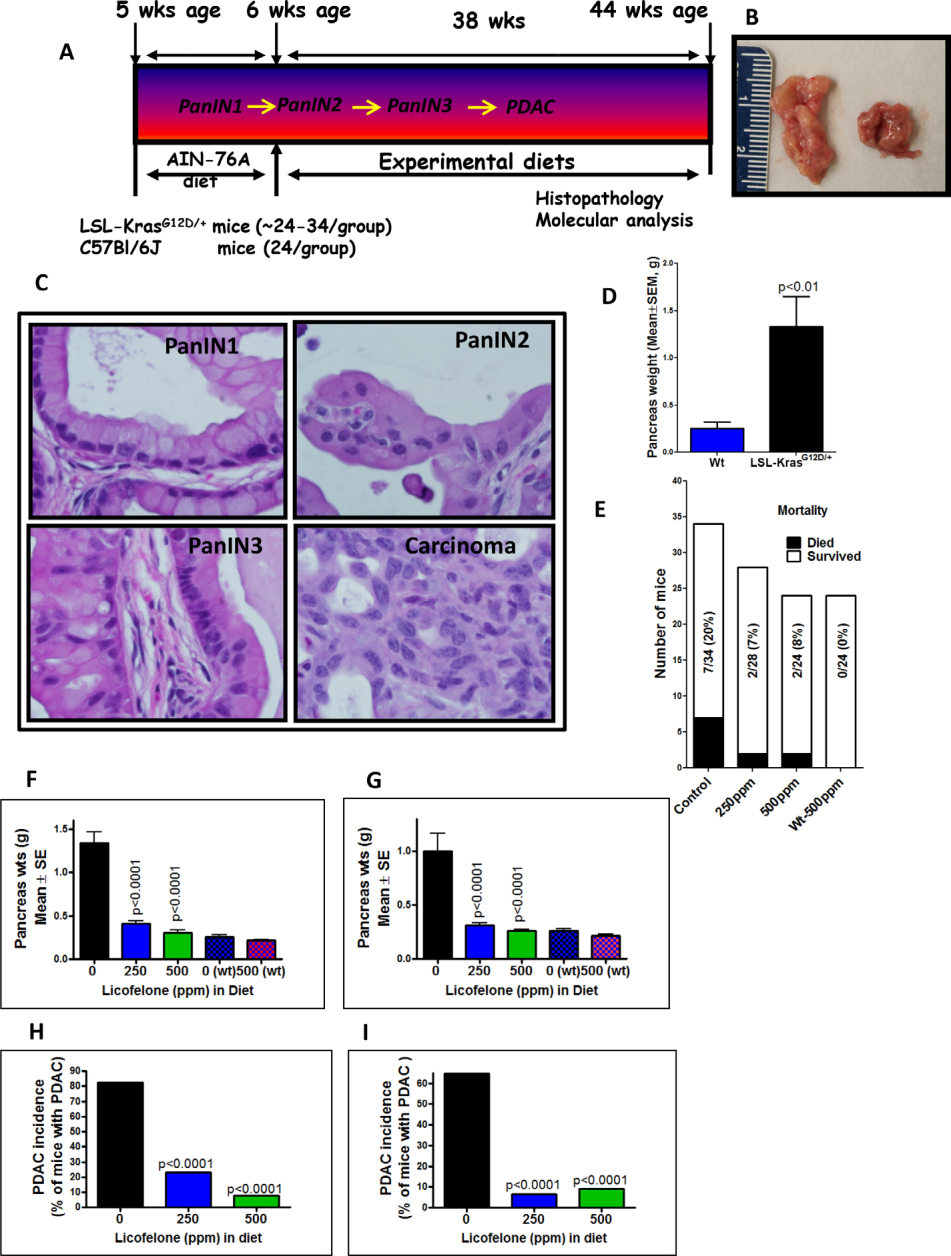
Evaluation of licofelone efficacy in pancreatic cancer prevention **A.** Experimental design for evaluation of licofelone efficacy in PC prevention in male and female p48^Cre/+^-LSL-Kras^G12D/+^ GEM mice. At 6-weeks of age, groups of mice (24–34/group for activated p48^Cre/+^-LSL-Kras^G12D/+^ or 24/group for wild-type) were fed AIN76-A diets containing 0, 250 or 500 ppm licofelone continuously for 38 weeks and each pancreas was evaluated histopathologically for marker expression as described in the text. **B–D.** Pancreatic tumor development in GEM. GEM (left) and wild type (right) pancreas **B.** PanIN lesions and carcinoma in GEM **C.** Pancreas weight at 44 weeks age in GEM compared to wild type (wt) mice (shown in parentheses) **D. E.** Effect of licofelone on mortality of GEM. **F–G.** Effect of licofelone on pancreas weight at the termination of the experiment in male **F.** and female **G.** mice. Both doses of licofelone significantly reduced the pancreatic tumor weights. **H–I.** Effect of licofelone on the incidence of PDAC in male **H.** and female **I.** mice.

To evaluate the effects of dual COX/5-LOX inhibition in a GEM model of PDAC on the inflammation acquired during tumor progression in the absence of cerulein treatment to mimic the natural scenario of disease progression in human, we used p48^Cre/+^-LSL-Kras^G12D/+^ mice, in which Cre recombinase activates the KrasG12D oncogene specifically in the pancreas. For this evaluation, mice were fed licofelone at 0, 250 and 500 ppm in the diet for 38 weeks beginning at 6 weeks of age (Fig. 4A). These mice develop initial pancreatic precursor lesions PanIN 1, 2 and 3 progressing to ductal adenocarcinoma with 100% penetrance as the mice age (12–18; Fig. 4B–4D). The average pancreas of wild type mice weighs about 0.26 g, whereas the pancreas (pancreatic tumor) of transgenic mice at 44 weeks of age weighs about 1.33 g, almost five-fold more (Fig. 4D). We examined PDAC development (Supplementary Fig. 2D–2E) and treatment effects of licofelone on the p48^Cre/+^-LSL-Kras^G12D/+^ mice. Mice that were euthanized before termination (due to sickness) or found dead constituted ~20% (7 of 34 mice) in the control group and ~7% (2 of 28 mice) and ~8% (2 of 24 mice) in 250 ppm and 500 ppm licofelone treatment groups, respectively (Fig. 4E). All wild type and transgenic mice fed control or licofelone diets had steady body weight gains (Supplementary Fig. 2F–2G). At 44 weeks of age, all mice were euthanized and evaluated for pancreatic weights (Fig. 4F–4G). None of the animals fed experimental diets exhibited any observable toxicity or any gross morphologic changes in liver, spleen, kidney or lung despite notable and significant differences in the pancreas weights. Licofelone treatment reduced the pancreas weights significantly in comparison with those of untreated transgenic mice (Fig. 4F–4G). Pancreas weights in the high dose licofelone group were almost equal to those of the wild type mice in both genders (Fig. 4F). We observed a 63–77% reduction in the pancreatic tumor weights in the treatment groups compared with the untreated control group (Fig. 4F–4G).

### Inhibition of PDAC incidence, PanIN lesions and carcinoma spread by licofelone

**Figure 5:**
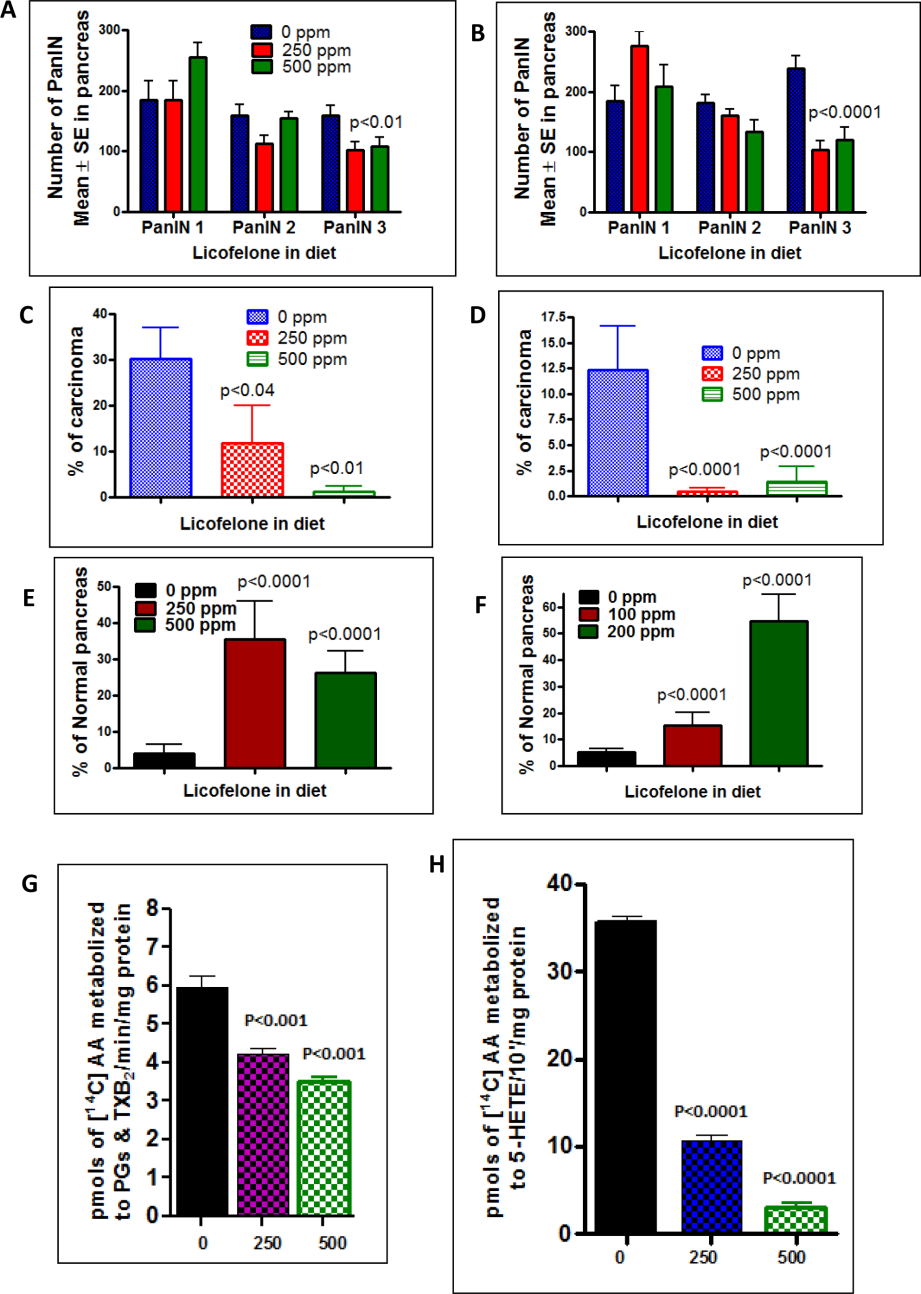
Inhibition of PDAC incidence, PanIN lesions, carcinoma spread and arachidonic acid metabolites by licofelone **A–B.** Effect of licofelone on PanIN multiplicity in male (panel A) and female (panel B) GEM (means ± SE). **C–D.** Effect of licofelone on the percentage of pancreas with carcinoma (C-male, D-female). **E–F.** Effect of licofelone on the percentage of normal appearing pancreas (E-male, F-female). The data in the panels were analyzed by unpaired ‘*t*’-test with Welch's correction; values are considered statistically significant at *p* < 0.05. **G.** Effects of licofelone on COX activity in pancreatic tumors from GEM as assessed with the radio-HPLC method. Values are means ± SEM, *N* = 6 per treatment group. A significant (*P* < 0.001) inhibition of AA metabolites (PGs and TXB2) was observed in pancreas of licofelone-treated mice compared with control mice. **H.** Effects of licofelone on 5-LOX activity in pancreatic tumors from GEM as assessed with the radio-HPLC method. Values are means ± SEM, *N* = 6 per treatment group. A significant (*P* < 0.0001) inhibition of the 5-LOX metabolite 5-HETE was observed in licofelone-treated mice compared with control mice.

Histological analysis revealed that 82% (14/17) of the untreated male and 65% (11/17) of untreated female mice had PDAC, whereas 3/13 male and 1/15 female mice treated with 250 ppm licofelone and only 1/13 male and 1/11 female mice treated with 500 ppm showed evidence of PDAC (Fig. 4H–4I). All the p48^Cre/+^-LSL-Kras^G12D/+^ mice with or without licofelone developed PanIN lesions. We observed 185 PanIN 1, 158 PanIN 2 and 158 PanIN 3 lesions in untreated males and 185 PanIN1, 181 PanIN2 and 238 PanIN 3 lesions in untreated females (Fig. 5A–5B). However, licofelone treatment caused a significant decrease in the development of PanIN 2 lesions and carcinoma *in situ* (PanIN 3 lesions) (Fig. 5A–5B). We then examined the pancreas for the spread of carcinoma. An average of 30.2% of untreated male mouse pancreas was occupied by PDAC. Pancreas from male mice treated with licofelone at 250 and 500 ppm showed carcinoma spread of only 12 and 1.3% or inhibition of 72 and 90%, respectively (Fig. 5C). The female mice treated with both doses of licofelone showed more than 96% inhibition of carcinoma spread (Fig. 5D). The normal-appearing pancreas of control mice comprised only 4% and 5% of the total pancreatic tissues in the males and females, respectively. After licofelone treatment, normal-appearing pancreatic tissue constituted a significantly higher percentage of the pancreas in both genders (Fig. 5E–5F).

### Licofelone modulates arachidonic acid metabolism

Licofelone exerts its effects by modulating COX-2- and 5-LOX-generated arachidonic acid metabolites. We measured total COX and 5-LOX activity by radiometric HPLC. A significant decrease was seen in total COX and 5-LOX metabolites in the pancreatic tumor tissue from mice treated with 250 and 500 ppm licofelone. As shown in Fig. 5G & 5H, mean total PG and thromboxane B2 (TXB2) generation in pancreas of control mice versus mice fed 250 or 500 ppm licofelone were 5.95 versus 4.22 and 3.51 pmoles/min; and 5-HETE was 35.68 versus 10.65 and 3.12 pmoles/min, respectively. Total COX and 5-LOX activities were reduced significantly in pancreatic tumor tissues from low and high dose licofelone-treated mice compared with tumors from control mice. Total COX was inhibited by 29.19% (*P* < 0.001) and 41% (*P* < 0.0001), and 5-LOX was inhibited by 70% (*p* < 0.0001) and 91% (*p* < 0.0001), respectively, with the low and high licofelone doses (Fig. 5G–5H). We also have noticed a significant decrease in *β*-catenin in both treatment groups (Supplementary Fig. 3A).

### Anti-inflammatory licofelone inhibits proliferation and induces apoptosis

**Figure 6:**
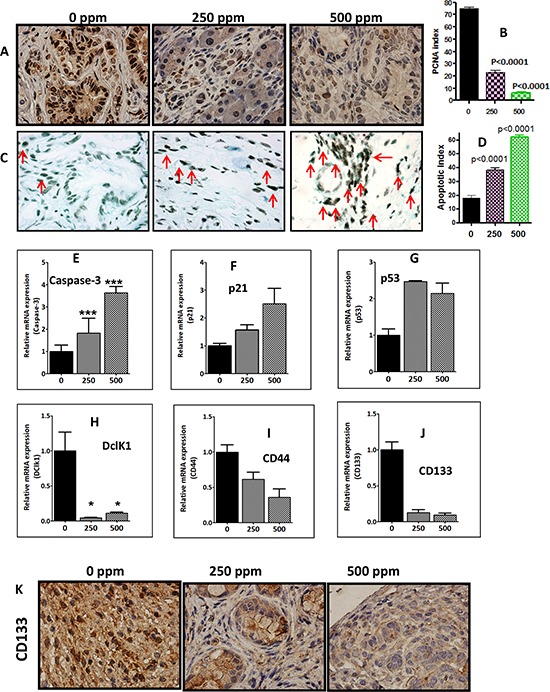
Anti-inflammatory licofelone inhibits proliferation, CSCs and induces apoptosis **A.** Immunohistochemical staining for PCNA in pancreatic tumors from GEM fed control diet or treated with licofelone. **B.** A significant difference was observed in the proliferative index between licofelone-treated and control group pancreas. **C–D.** TUNEL assay was done for apoptotic cells in pancreatic tumors from GEM fed control diet or treated with licofelone (*N* = 6 mice/group). A significant induction of apoptosis was observed in treated mice compared with untreated mice tumors. **(E–J)** Relative mRNA expression of apoptosis (caspase-3, p21 and p53) and CSC markers (DclK1, CD44, CD133) as determined by real time PCR. Licofelone treatment significantly increased expression of caspase-3 **E.** p21 **F.** and p53 **G.** and decreased expression of CSC markers DclK1 **H.** CD44 **I.** and CD133 **J. K.** Imunohistochemical staining showing a decrease in CD133 expression in the treatment groups.

Fig. 6A and 6B summarize the effects of licofelone on tumor cell proliferation as measured by PCNA overexpression. Quantification of PCNA staining showed 75 ± 1.34 (mean ± SEM) PCNA-positive cells in pancreas from untreated, as compared with 23 ± 1.54 and 6 ± 1.25 PCNA-positive cells in low and high dose licofelone-treated mice, accounting for a decrease in the proliferation index of approximately 69% and 92% (*P* < 0.0001), respectively (Fig. 6B). Fig. 6C and 6D summarize the effects of licofelone on tumor cell apoptosis. Qualitative microscopic examination showed a substantial increase in TUNEL-positive cells in the pancreatic tumor tissue of mice treated with licofelone. Quantification of TUNEL-positive cells from pancreas of control diet-fed mice showed 18 ± 1.8 (mean ± SEM) as compared with 38 ± 1.63 and 62 ± 1.33 TUNEL-positive cells in pancreatic tumor tissue from licofelone-treated mice, accounting for an increase in the apoptotic index of more than 52% and 71% (*P* > 0.0001), respectively (Fig. 6D). In agreement with the apoptosis results, we observed a significant increase in Caspase-3, p21 and p53 mRNA expression (Fig. 6E–6G) and a decrease β-catenin in the treatment groups (Supplementary Fig. 3A).

### Pharmacological inhibition of inflammation by licofelone Inhibits cancer stem cells in GEM

**Figure 7:**
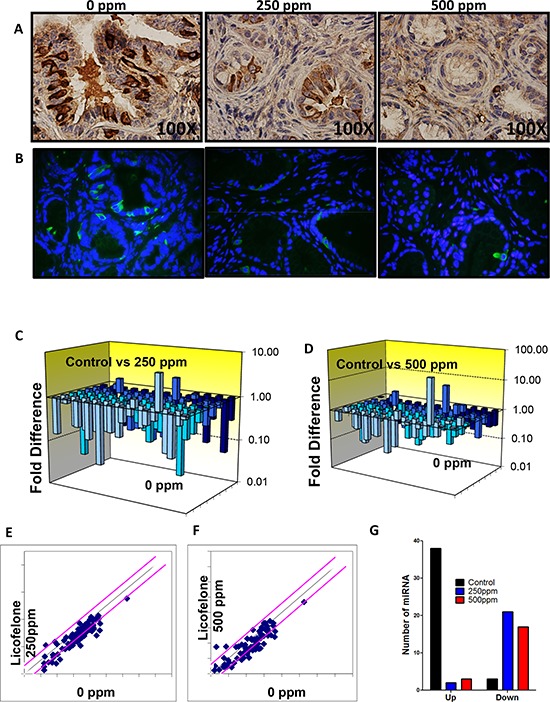
Licofelone inhibits DclK1 and modulates miRNAs associated with inflammation and cancer stem cells **A–B.** Effect of licofelone on DclK1. Immunohistochemical (brown-DclK1) and Immunofluorescence (green-DclK1, blue-DAPI) staining for DclK1 in pancreatic tumors from GEM fed control diet or treated with licofelone. A significant dose-dependent decrease was observed in Dclk1 expression in licofelone-treated group pancreas. **C–G.** Effect of licofelone on miRNAs including those related to inflammation and CSCs as determined by PCR arrays by real time pcr.

We previously have shown that DclK1 is a potential stem cell marker in pancreatic carcinogenesis (34). By inhibiting COX-2, 5-LOX, cytokines and their receptors, anti-inflammatory agents can block signals from tumor cells, and potentially affect CSCs. We have seen a significant decrease in expression of DclK1 along with other CSC markers (CD133, Lgr5, CD166) and in their co-localization upon licofelone treatment (Fig. 6H–6K). Licofelone treatment significantly reduced the expression of DclK1 in dose-dependent manner (Fig. 7A–7B). Quantification of DclK1-positive cells in pancreatic tumor from control diet-fed mice showed 80 ± 1.34 (mean ± SEM), as compared with 45 ± 1.54 and 30 ± 1.25 in tissue from mice treated with low or high dose licofelone, for a decrease in cancer stem cells by 43% and 62% (*P* > 0.0001), respectively (Supplementary Fig. 3B–3C). Co-expression of Dckl-1 with other stem cell markers also was reduced in the treatment groups (Supplementary Fig. 4). *In vitro*, licofelone caused a dose-dependent inhibition of cell viability, colony forming units and inhibited stem cell and inflammatory markers (Supplementary Fig. 5). Collectively, these results show the potential of licofelone to delay progression of pancreatic lesions to carcinoma via multiple effects.

### Modulation of miRNA by licofelone

To determine whether inflammatory and CSC miRNAs are altered concurrently we used, the Mouse Cancer PathwayFinder™ RT^2^ Profiler™ PCR Array from SA biosciences to study the expression of 84 genes representative of six biological pathways involved in transformation and tumorigenesis. Many pancreatic tumor tissue miRNAs were altered significantly in response to treatment of mice with licofelone. In untreated pancreatic tumors from the GEM mice, 38 miRNAs were induced while 3 were suppressed compared with normal pancreas from wild type mice (Fig. 7C–7G, Supplementary Table 2). Licofelone treatment suppressed 21 of the induced miRNAs, and significantly induced the 3 miRNAs that were suppressed in the untreated group by two-fold or greater (Fig. 7C–7G, Supplementary Table 2). Most of these altered miRNAs regulate inflammatory signaling, which is regulated significantly by licofelone treatment. Most importantly, the miRNAs most strongly implicated in regulation of arachidonic acid metabolism via COX-2 and 5-LOX — like miR-199a, miR-21, miR-146, miR-29, miR148 — were tremendously modulated by licofelone treatment, clearly demonstrating targeted effects of this agent (Fig. 7C–7G, Supplementary Table 1). These results are consistent with alteration by licofelone of mRNA, protein and activity levels of the miRNA-targeted inflammatory pathway genes. Similarly, miRNAs — like miR-140, miR-150, miR-122 and miR-31 — that regulate cancer stem cell genes were suppressed significantly by licofelone treatment.

## DISCUSSION

Treatment of PC has been a big unmet challenge. The aim of this study was to shed light on the role of inflammation in DclK-1-positive CSCs and pancreatic adenocarcinoma and to evaluate the pharmacologic effects of licofelone on PC. The existence and the levels of CSCs have profound implications for cancer treatment due to the likelihood that eradication of CSCs is the critical determinant in achieving ultimate cure [34, 35]. We have demonstrated that DclK-1 (DCAMKL-1), is a novel putative pancreatic stem cell marker and identified DclK-1 as potent target for pancreatic tumor eradication. Our laboratory and others have shown overexpression of the markers DclK-1, CD-44 and CD-133, representing cancer stemness, in PC [17, 34–36]. DCLK1 mark morphologically distinct and functionally unique population of pancreatic cancer-initiating cells. Cells that expressed DCLK1 expressed high levels of ATAT1, HES1, HEY1, IGF1R, ABL1, and manipulation of these pathways in PDAC cell lines inhibited their clonogenic potential [37]. Many PC treatment strategies result in tumor shrinkage but often fail to prevent recurrence due to their ineffectiveness against CSCs. Thus, novel prevention and treatment strategies that specifically target the CSC population may be more effective in obliterating this deadly disease. In this study, we determined whether inflammation plays a role in over-expression of DclK1 and whether the anti-inflammatory agent licofelone contributes to PC inhibition by modulating CSC markers. We found Inflammation in the early stages of pancreatic tumor progression (Supplementary Fig. 1) and we also observed DclK1 expression as early as 2 months of age in the PDAC GEM. DclK1 was expressed in both low grade and high grade PanIN lesions (PanIN 1, −2, −3). Both DclK1 and COX-2 expression was high in the human PDAC (Fig. 1I & 1J). These results suggest that CSCs get activated at an early stage along with inflammation and progresses in a linear way as the disease progresses. These results were corroborated with next generation sequencing of pancreas from wild type and GEM mice, which showed that DclK1, COX-2 and 5-LOX were increased significantly in tumors (Fig. 1).

Genetic ablation of COX-2 to decrease inflammation in GEM mice at 4 weeks of age led to a decrease in the DclK1 expression (Fig. 2A–2B). Previous reports have shown that decrease in COX-2 inhibits PC [15, 21]. To further demonstrate the role of inflammation on DclK1, we induced inflammatory changes in the pancreas of GEM with cerulean. We observed significantly enhanced pancreatitis along with increased PanIN lesions and increased DclK1 in cerulean-treated GEM. Licofelone dramatically inhibited inflammation, proliferation, thereby pancreatitis as well as PanIN lesions and DclK1 expression (Fig. 2C–2K & 3). These data support the conclusion that inflammation plays a role in regulating DclK1. Among dual COX/LOX inhibitors, licofelone is in advanced phase of clinical trials as an anti-inflammatory drug [38], and its safety and efficacy, in comparison with the non-steroidal anti-inflammatory drugs (NSAIDs) such as naproxen and rofecoxib, have been well documented [30, 39, 40].

Given these results and as rationalized in introduction, simultaneously targeting COX and 5-LOX pathways early during inflammation may be an effective way to suppress pancreatic tumor progression without unwanted cardiovascular and gastrointestinal side effects. In our long term *in vivo* efficacy study, we demonstrated that licofelone can target arachidonic acid metabolites derived from COX-2 and 5-LOX, cancer stem cell markers (DclK1, CD133, CD44 and Lgr5) and specific miRNAs and their target genes and can potently inhibit PanIN progression to PDAC without any unwanted side-effects. We found that licofelone inhibits COX-2 and 5-LOX activities, thus blocking production of tumorigenic mediators like PGE2. Licofelone-fed mice showed dramatic inhibition of PDAC incidence in both males and females. Development of carcinoma *in situ* (PanIN 3 lesions) was blocked by 35–56%. The most striking finding was that carcinoma spread was inhibited by > 95% in treatment groups, with a significant decrease in mortality of the mice. These results are consistent with our earlier studies on licofelone in colon cancer [30]. There are limited reports on evaluation of COX-2 inhibitors and no reports on dual COX/5-LOX inhibitiors in PC using a GEM model. For example, Funahasi *et al*. reported that the selective COX-2 inhibitor nimesulide delays the progression of PC precursor lesions in the KrasG12D mouse model [21].

Of note, licofelone effects on pancreatic tumor weights correlated very well with effects on PDAC incidence, carcinoma *in situ* development and invasiveness of cancer. Previous investigations of our group and others revealed anti-proliferative and apoptotic effects of licofelone on colon cancer [30, 41]. Our results here clearly indicate that licofelone effectively suppressed the growth and progression of PC in GEM. We propose that the synergism obtained due to simultaneous inhibition of COX and 5-LOX at early stages might be an underlying mechanism for the enhanced antitumor effects [30]. However, further studies are warranted to establish cause and effect.

In the present study, licofelone significantly suppressed increases in DclK1 along with other CSC markers (Lgr5, CD133 and CD44) in correlation with its inhibition of tumor progression, suggesting that licofelone may kill CSCs. Drugs like metformin have been shown to target specifically cancer stem cells, thereby showing greater effects in blocking pancreatic and breast tumor growth and prolonging remissions [17, 36, 42]. Real time PCR analysis showed deregulation of several miRNAs with oncogenic and tumor suppressor activities in the untreated pancreatic tumors. The link between aberrant miRNA expression and PC development suggests that miRNAs could be potential targets for chemopreventive and chemotherapeutic agents. In the present study, we showed that the expression of several miRNAs is altered during the development of PC and that licofelone reverses the altered expression of the majority of these miRNAs with up-regulation of miR-21, miR-222, Let-7, miR-125, miR-142 and down-regulation of miR-1, miR-122 and miR-148. In recent years, researchers have attempted to alter the expression of miRNAs for inhibition of cancer growth using several agents [43]. All of the miRNAs we studied are aberrantly expressed in human cancers. For example, Lee EJ 2007 *et al*. [44] showed that the miRNAs miR155, miR21, miR222, Let7, miR376a, miR301, miR100, miR125, miR142 and others are overexpressed significantly in human PC. Researchers also have found that miR-1 is down-regulated in several types of cancers [45–48] and that it acts as a tumor suppressor. MicroRNA-148a is down-regulated in human PDAC and regulates cell survival by targeting CDC25B [49]. Licofelone dramatically down-regulated the majority of miRNAs overexpressed in association with pancreatic tumor progression and upregulated miR1, miR122 and miR158 by many fold including those that regulate inflammation and CSCs. These results clearly demonstrate the ability of licofelone to regulate inflammation, CSCs and miRNAs in correlation with its inhibition of PC progression.

## MATERIALS AND METHODS

### Mouse model, diet and handling

All animal research was performed under the auspices of animal protocols approved by the University of Oklahoma Health Sciences Center institutional animal care and use committee. Animals were housed in ventilated cages under standardized conditions (21°C, 60% humidity, 12-h light/12-dark cycle, 20 air changes/hour) in the University rodent barrier facility. Semi-purified modified AIN-76A diet ingredients were purchased from Bioserv, Inc., NJ. Generation of p48^Cre/+^-LSL-Kras^G12D/+^ mice expressing the activated KrasG12D oncogene has been described previously [17, 18]. The GEM KrasG12D (LSLKras/Ela-CreERT mice) alone or crossed with COX2 conditional knockout mice (COXKO/LSL-Kras/Ela-CreERT) were used to study the effect of ablation of COX-2 on DclK1. The dual COX/5-LOX inhibitor Licofelone was procured from the NCI-DCP chemoprevention drug repository. Mice were allowed *ad libitum* access to the respective diets (Licofelone 250 and 500 ppm) and to automated tap water purified by reverse osmosis.

### Cerulein-induced inflammation in GEM mice

After genotyping, pancreatitis was induced by injecting cerulein intraperitoneally (i.p.) in twelve-week-old p48^Cre/+^-LSL-Kras^G12D/+^ mice (*n* = 6/group) with or without licofelone according to the published method with modification [50]. Briefly, all of the mice were injected with 5 μg/mice of cerulein every day for six consecutive days. Licofelone (500 ppm) was fed to a group of mice starting one day before cerulein injection to one day after the last cerulein injection. One day after cerulein injection, all mice were euthanized by CO2 asphyxiation and necropsied. Pancreata were collected from all groups, weighed and snap frozen in liquid nitrogen for further analysis. Pancreata required for histopathology (to evaluate pancreatitis and PanINs) and immunohistochemistry (IHC) for evaluation of various molecular markers were fixed (head to tail) in 10% neutral-buffered formalin as previously described [12–18].

### Tissue processing and histological analysis of pancreatitis and PanIN lesions

After euthanzing the mice, pancreata and other key organs (including liver, spleen, kidney, lung) were collected and weighed. Tissues were fixed in 10% formalin for 24 h and routinely processed and embedded in paraffin. Pancreatitis was analyzed and graded using a semi-quantitative scoring system according to established criteria in the literature by a pathologist blinded to the treatment group. Briefly, the pancreatitis index was expressed as a sum of scores on severity of pancreatitis, loss of acini, extent of inflammatory cell infiltration and stromal fibrosis as per earlier studies [33, 50]. To quantify the progression of PanIN lesions, the total number of ductal lesions and their grades were determined [12–18].

### Preclinical assay: efficacy of licofelone

Genotyped male and female p48^Cre/+^-LSL-Kras^G12D/+^ transgenic mice were used in the efficacy study. Five week-old mice were selected and randomized so that average body weights in each group were equal and were fed AIN-76A diet for one week. At 6 weeks of age, p48^Cre/+^-LSL-Kras^G12D/+^ mice were fed AIN-76A experimental diets containing 0 ppm (*n* = 34/group + *n* = 12 C57BL/6 wild-type mice), 250 ppm (*n* = 28/group) or 500 ppm *n* = 24/group licofelone in the diet until termination of the study. After 38 weeks (~10 months) on experimental diets, all mice were euthanized by CO_2_ asphyxiation and necropsied; pancreata were collected from all groups, weighed and snap frozen in liquid nitrogen for further analysis. Pancreata (head to tail) required for histopathologic and IHC evaluations to identify PanIN lesions and PDAC for evaluation of various molecular markers were fixed in 10% neutral-buffered formalin as previously described.

### Histological analysis of panIN lesions and PDAC

Formalin fixed tissue sections (4 μm) of each pancreas stained with Hematoxylin & Eosin (H&E) were histologically evaluated by a pathologist blinded to the experimental groups. PanIN lesions and carcinoma were classified according to histopathologic criteria as recommended elsewhere [4, 12–18]. Similarly, pancreatic carcinoma and normal appearing pancreatic tissue were evaluated in all animals.

### Immunohistochemistry and immunofluorescence

5 μm fixed sections were incubated with primary antibodies in a hybridization chamber for 1 h at room temperature or overnight at 4°C. The primary antibodies used were DclK1, COX-2, 5-LOX, Ki67, proliferating cell nuclear antigen (PCNA), CD133, CD44, Lgr5, Annexin V and β-catenin procured from Santa Cruz/Abgent/Abcam/Abcam/Cell Signaling. Following primary antibody, sections were incubated for 1 h with anti-mouse/anti-rabbit/anti-goat secondary antibody, then visualized with diaminobenzidine (DAB) and counterstained with H&E for IHC or with DAPI for immunohistofluorescence (IHF). Slides were observed under an Olympus microscope 1X701 and digital computer images were recorded with an Olympus DP70 camera.

### Analysis of COX-1 and -2 activity using Radio-high performance liquid chromatography (HPLC)

Frozen pancreatic tumor tissues from male mice fed 250 or 500 ppm of licofelone or control diet were homogenized using ice-cold homogenizing buffer. For COX-1 and COX-2 assays, 150 μL of reaction mixture containing 12 μmol/L [14C] arachidonic acid (AA; 420, 000 dpm), 1 mmol/L epinephrine, 1 mmol/L glutathione in 50 mmol/L phosphate buffer (pH 7.4) were incubated with 30 mg of lysate protein at 37°C for 20 minutes. The reactions were terminated by adding 40 μL of 0.2mol/L HCl. The COX-mediated metabolites of AA were extracted with ethyl acetate (3 × 0.5 mL). The combined extracts were evaporated to dryness under N2, dissolved in 1 mL of acetonitrile and 10 μL were injected into a reverse phase HPLC system (Shimadzu Scientific Instruments, USA) equipped with a Phenomenix C18 column (300 × 3.90 mm; pore size 10 μ). The [14C]-PGs, [14C]-TxB2 and [14C]-PGE2 were eluted with a gradient solvent system containing solvent A: Acetonitrile:Water:Acetic acid (35:65:0.1%) and solvent B: Acetonitrile:Water:acetic acid (65:30:0.1%). The eluted metabolites were monitored and quantified with an IN/US Systems β-RAM radio HPLC detector.

### Apoptosis assay

Paraffin sections (*N* = 6 mice/group) of 5-μm thickness mounted on slides were rehydrated and stained using the Fragment End Labeling (FragEL) DNA Fragmentation Detection Kit with the terminal deoxynucleotidyl transferase dUTP nick end labeling (TUNEL) method following the manufacturer's instructions (Millipore, Billerica, MA). This kit allows the recognition of apoptotic nuclei in paraffin-embedded tissue sections fixed on slide by FragEL of DNA. The terminal deoxynucleotidyl transferase binds to exposed ends of DNA fragments generated in response to apoptotic signals and catalyzes the template-dependent addition of biotin-labeled and biotin-unlabeled deoxynucleotides. Biotinylated nucleotides are detected using streptavidin-HRP conjugate. Diaminobenzidine reacts with the labeled sample to generate an insoluble colored product at the site of DNA fragmentation. Counter-staining with methyl green aids in the morphologic evaluation and characterization of normal and apoptotic cells. Stained apoptotic epithelial cells (a minimum of 10 microscopic fields per section) were counted manually in a single-blind fashion.

### Quantitative real-time PCR for miRNA analysis

The RT2 miRNA PCR Array Mouse miRNA Panel v1.7 (SABiosciences, Frederick, MDA, USA) was employed for miRNA profiling. The RT2 qPCR-Grade™ miRNA Isolation Kit (SA Biosciences) was used to enrich small RNA. The RT2 miRNA First Strand Kit was used to convert small RNA into cDNA. Reverse transcription (RT) was performed at 37°C for 2 h by incubating a 10 ul mixture including 400 ng of small RNA, 5X miRNA RT primer, 1ul ERC mix, 2ul reverse transcription buffer, 1ul RT enzyme mix, and nuclease-free water. The quantitative real-time polymerase chain reaction (PCR) mixtures, consisting of 100 ul of diluted first reaction, 1.275 mL 2X RT2 SYBR master mix (SABiosciences, Frederick, MDA, USA), and 1.175 ul nuclease-free water, were incubated for 40 cycles on a Bio-Rad IQ-5 real-time PCR System (Bio-Rad, Hercules, CA, USA). Each cycle included denaturing for 15 s at 95°C, annealing and extension for 30 s at 60°C, and 30 s at 72°C. The comparative Ct method was used to compute relative levels of target miRNAs by subtracting the Ct values of the endogenous control. Samples were reported as n-fold relative to the calibrator set at 1.0.

### Statistical analysis

The data are presented as means ± SE. Differences in body weights were analyzed by *ANOVA*. Statistical differences between control and treated groups were evaluated using Fisher's exact test for PDAC incidence and unpaired *t*-test with Welch's correction was used for PanIN and PDAC lesions. Differences between groups are considered significant at *p* < 0.05.

## CONCLUSION

These results offer a preclinical proof of concept to target the inflammation to inhibit cancer stem cells early for improving the treatment of pancreatic cancers, with immediate clinical implications for repositioning dual COX/5-LOX inhibitors in human trials for high risk patients.

## SUPPLEMENTARY FIGURES AND TABLES


