# Supplementary material for: Targeting pancreatitis blocks tumor-initiating stem cells and pancreatic cancer progression
Source: Oncotarget. 2015 Mar 26;6(17):15524–39. doi: 10.18632/oncotarget.3499 (PMC4558168; doi:10.18632/oncotarget.3499)
Supplement: Supplementary file 1 [file oncotarget-06-15524-s001.pdf]

SUPPLEMENTARY FIGURES AND TABLES

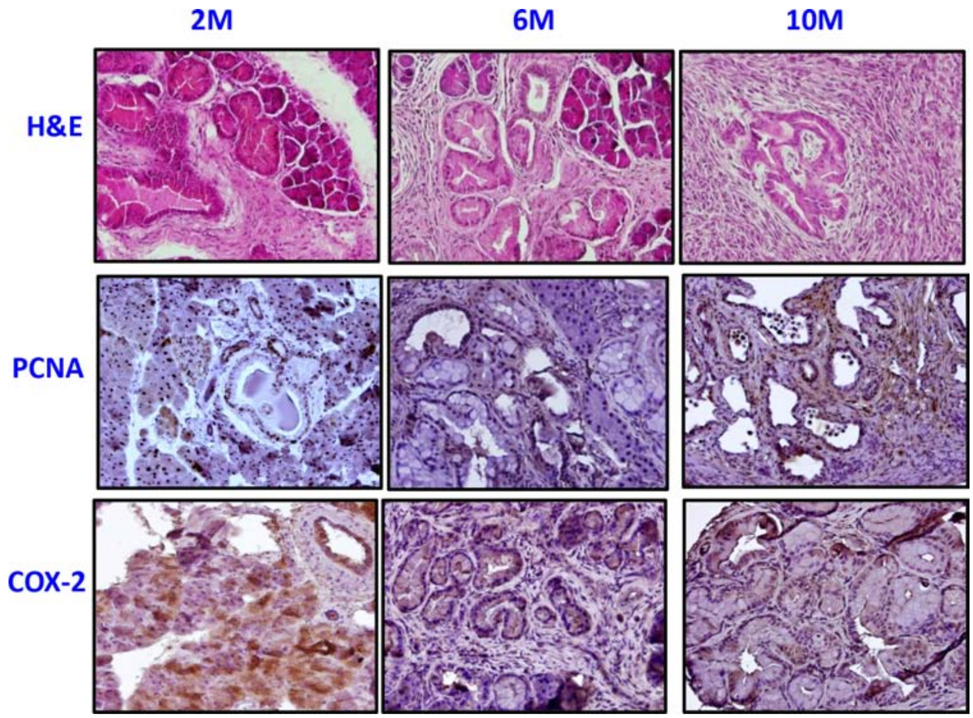

Supplementary Figure S1: Hematoxylin and Eosin, PCNA and COX-2 immunohistochemical staining of pancreas from −2, −6 and 10 months old GEM. PanIN lesion progression and formation of carcinoma increases as the mice age with increase in PCNA and COX-2 expression.

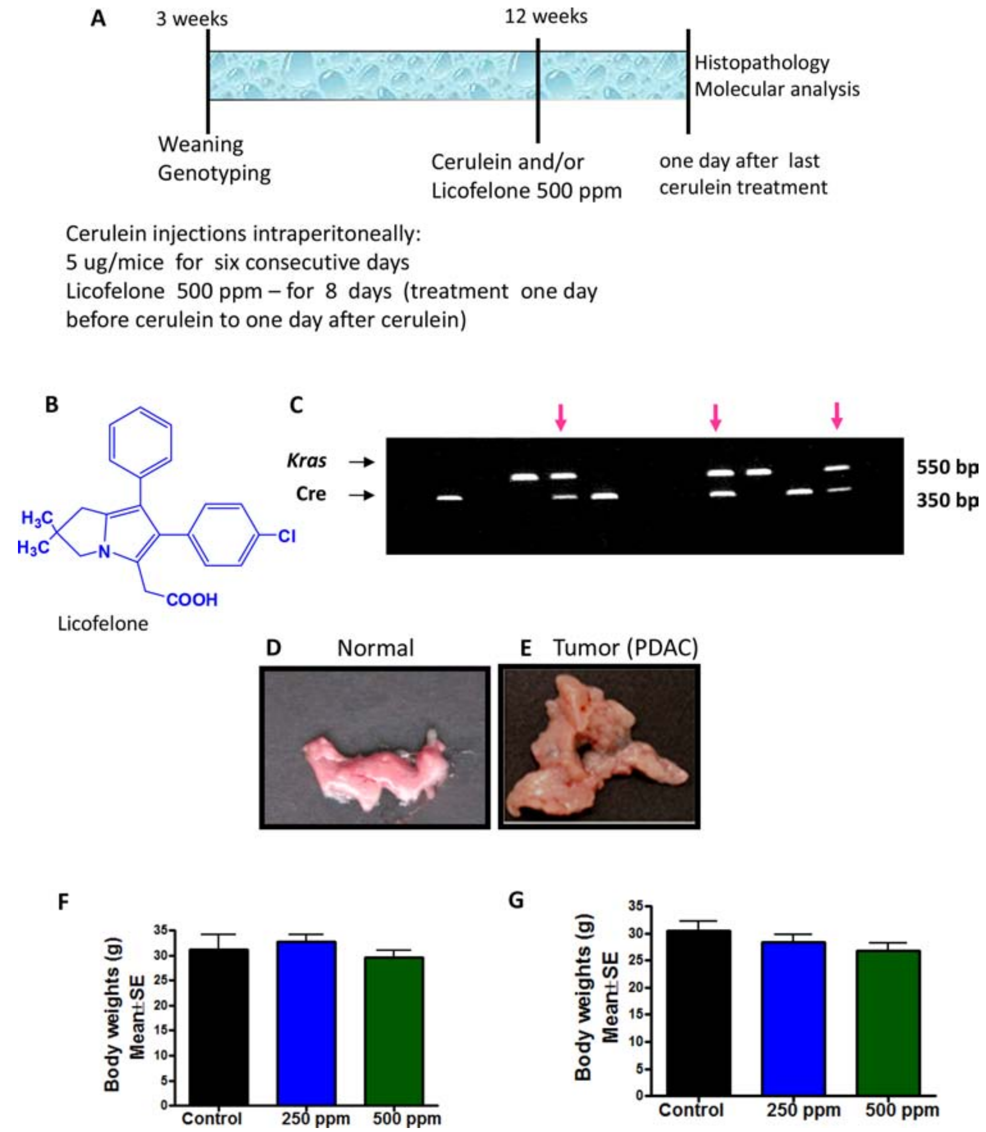

Supplementary Figure S2: (A) Experimental design for cerulein and licofelone treatment of GEM. At 12-weeks of age, groups of mice (6/group) were injected with cerulein as described in the methods. **(B)** Structure of licofelone. **(C)** Representative genotyping result for Kras$^{G12D/+}$ and p48$^{Cre/+}$ mice. **(D-E)** Photograph showing normal pancreas (D) and PDAC (E) **(F-G)** Effect of licofelone on body weight (BW; means ± SE) at termination of the experiment. No statistically significant difference was observed between control and licofelone-treated male (E) and female (F) GEM mice.

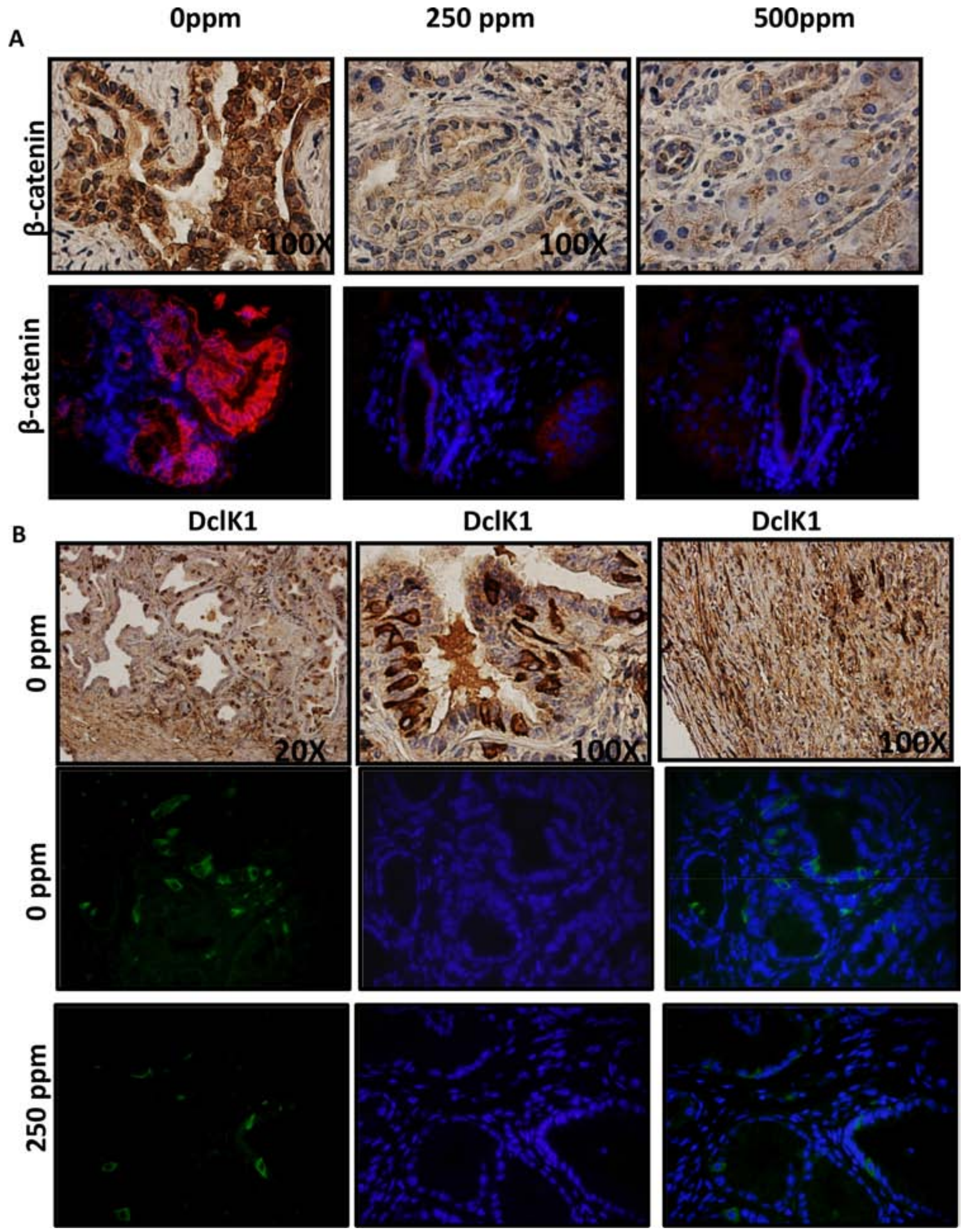

Supplementary Figure S3: Effect of licofelone on β-catenin and DclK1 expression as determined by immunohistochemistry (brown- β-catenin and DclK1) and immunofluorescence (red-β-catenin, gren-DclK1 and blue-DAPI) staining. A significantly decreased β-catenin and DclK1 expression was observed upon licofelone treatment.

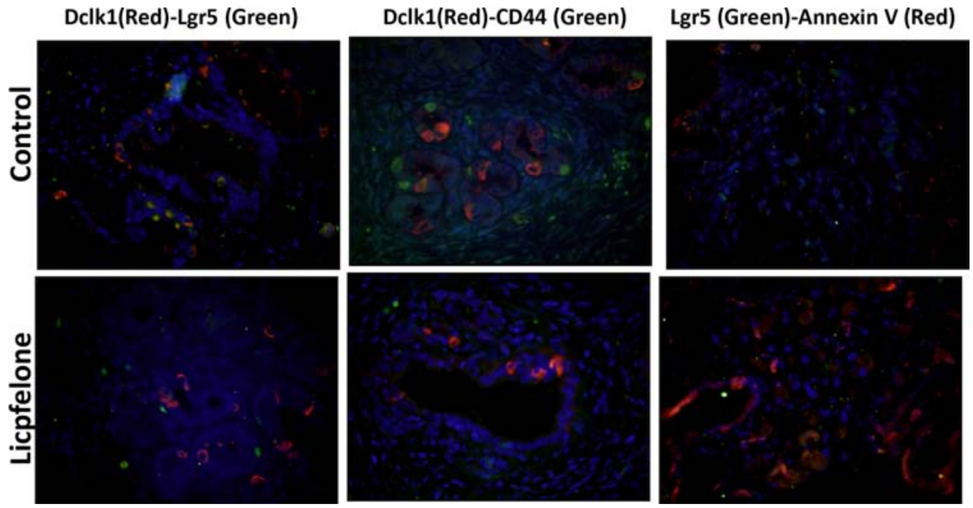

Supplementary Figure S4: Effect of licofelone on co-expression of CSC (DclK1, Lgr5 and CD44) and apoptosis (Annexin V) markers in pancreatic tumors. Immunofluorescence analysis was performed on paraffin-embedded and micro-sectioned pancreatic tissues as described in the Methods section. A significantly decreased co-expression of DclK1/Lgr5 and DclK1/CD44 ratio was seen with licofelone treatment. Decreased Lgr5 expression with increased annexin V expression was observed in the licofelone-treated GEM pancreas.

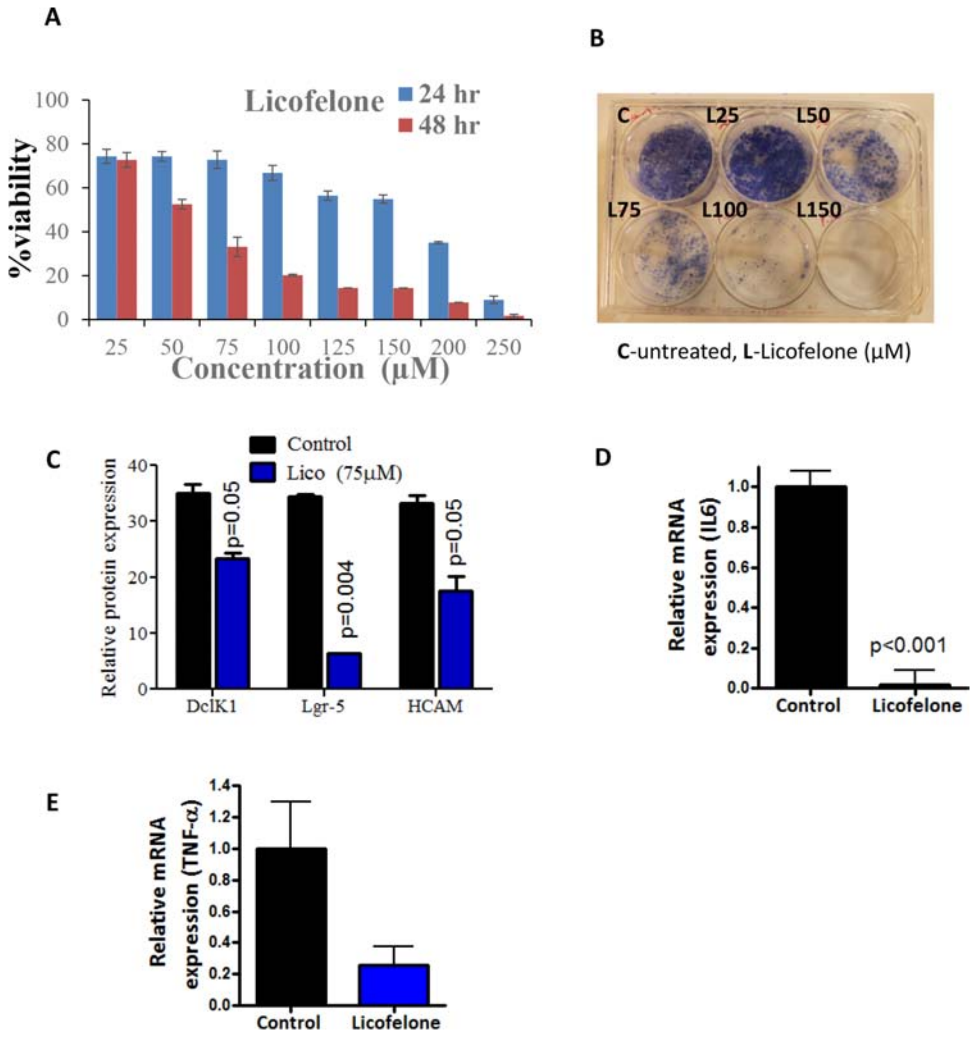

Supplementary Figure S5: (A-B) Effect of licofelone on viability (A) and colony formation ability (B) in MiaPaCa cells. (C) Effect of licofelone on CSC markers. **(D-E)** Effect of licofelone on IL6 (D) and TNFα (E).

Supplementary Table S1. Table showing the pancreatitis scoring

Pancreatitis	Stromal Fibrosis
1. Slight	1. <5%
2. Mild	2. 5–10%
3. Modest	3. 10–20%
4. Severe	4. >20%
**Loss of Acini/Acinar destruction**	**Inflammatory cell infiltration**
1. <10%	1. 1–30 cells
2. 10–30%	2. 31–50 cells
3. 30–50%	3. 51–100 cells
4. >50%	4. >100 cells

Supplementary Table S2. Expression of miRNAs in normal pancreas vs pancreatic tumors at 44 weeks age as determined by real time pcr arrays. Effect of licofelone on miRNA changes compared with untreated pancreatic tumors

	Normal vs tumor		Tumor vs 250 ppm		Tumor vs 500 ppm	
	Fold change	*p* Values	Fold change	*p* Values	Fold change	*p* Values
miR-125	27.97	0.004	−3.99	0.0002	−13.82	0.0001
miR-142	21.35	0.005	−23	0.0007	−4.54	0.0005
miR-96	8.48	0.01	−6.94	0.0009	−6.86	0.0005
miR-222	5.14	0.01	−4.02	0.0006	−3.35	0.03
miR-205	174.64	0.001	−5.86	0.0001	−10.92	0
miR-199	32.05	0.05	−5.45	0.0009	−12.42	0.01
miR-140	61.42	0.008	−4.75	0.02	−4.1	0.02
miR-183	17.82	0.03	−3.86	0.01	−4.17	0.01
miR-34c	35.66	0.008	−7.1	0.0004	−5.96	0.0004
miR-29b	8.42	0.04	−38.85	0.0002	−7.82	0.006
miR-135b	129.85	0.009	−5.08	0.01	−12.87	0.003
miR-21	126.66	0.01	−5.32	0.02	−3.74	0.03
miR-18a	16.69	0.01	−7.83	0.001	−3.74	0.0005
miR-214	15.94	0.01	−7.54	0.0005	−3.34	0.003
miR-27a	44.79	0.04	−3.32	0	−5.65	0.0002
miR-130a	7.73	0.03	−5.29	0.002		
miR-146b	98.36	0.02	−3.64	0.009		
miR-363	4.09	0.01	−7.52	0.002		
miR-150	123.16	0.004	−9.68	0.0008		
miR-Let7i	2.08	0.005	−4.24	0		
miR-19a	6.46	0.03	−4.47	0.04		
miR-106	6.46	0.01			−3.24	0.001
miR-31	62.37	0.0007			−8.73	0.0002
miR-122	−14.54	0.03	6.14	0.0008	27.74	0.002
miR148	−9.56	0.02	3.06	0.15	8.04	0.04
miR1	−7.68	0.09			2.06	0.05
